# Effect of siponimod on lymphocyte subsets in active secondary progressive multiple sclerosis and clinical implications

**DOI:** 10.1007/s00415-024-12362-9

**Published:** 2024-04-17

**Authors:** Antonio Luca Spiezia, Giulia Scalia, Maria Petracca, Daniele Caliendo, Marcello Moccia, Antonia Fiore, Vincenza Cerbone, Roberta Lanzillo, Vincenzo Brescia Morra, Antonio Carotenuto

**Affiliations:** 1 Multiple Sclerosis Clinical Care and Research Centre, Department of Neuroscience, Reproductive Science and Odontostomatology, Federico II University of Naples, Via Sergio Pansini 5, 80131 Naples, Italy; 2 Clinical and Experimental Cytometry Unit, Centre for Advanced Biotechnology Franco Salvatore, CEINGE, Naples, Italy; 3 Department of Human Neurosciences, Sapienza University, Rome, Italy; 4 Department of Molecular Medicine and Medical Biotechnology, Federico II University of Naples, Naples, Italy

**Keywords:** Multiple sclerosis, Siponimod, Progression, Lymphocytes, Prognosis

## Abstract

**Background:**

Circulating immune cells play a pathogenic role in multiple sclerosis (MS). However, the role of specific lymphocyte subpopulations is not unveiled yet, especially in progressive stages. We aimed to investigate lymphocyte changes during siponimod treatment in active secondary progressive MS (aSPMS) and their associations with clinical outcomes.

**Methods:**

We enrolled 46 aSPMS patients starting on siponimod treatment with at least 6 months of follow-up and two visits within the scheduled timeframes and 14 sex- and age-matched healthy controls (HCs). Clinical and laboratory data were collected retrospectively at baseline, 3rd, 6th, 12th, and 24th month for MS patients, and at baseline for HCs.

**Results:**

At baseline SPMS patients presented with increased naïve regulatory T lymphocytes (*p* = 0.02) vs. HCs. Over time, SPMS patients showed decreased T CD4+ (coeff. range = −24/−17, 95% CI range = −31.60 to −10.40), B lymphocyte (coeff. range = −3.77/−2.54, 95% CI range = −6.02 to −0.35), memory regulatory B cells (coeff. range = −0.78/−0.57, 95% CI range = −1.24 to −0.17) and CD4/CD8 ratio (coeff. range = −4.44/−0.67, 95% CI range = −1.61 to −0.17) from month 3 thereafter vs. baseline, and reduced CD3+CD20+ lymphocytes from month 12 thereafter (coeff. range = −0.32/−0.24, 95% CI range = −0.59 to −0.03). Patients not experiencing disability progression while on siponimod treatment showed B lymphocyte reduction from month 3 (coeff. range = −4.23/−2.32, 95% CI range = −7.53 to −0.15) and CD3+CD20+ lymphocyte reduction from month 12 (coeff. range = −0.32/−0.24, 95% CI range = −0.59 to −0.03) vs. patients experiencing progression.

**Conclusions:**

Patients treated with siponimod showed a T and B lymphocyte reduction, especially CD4+, CD3+CD20+ and naïve regulatory T cells and memory regulatory B cells. Disability progression while on siponimod treatment was associated with a less pronounced effect on B and CD3+CD20+ lymphocytes.

**Supplementary Information:**

The online version contains supplementary material available at 10.1007/s00415-024-12362-9.

## Introduction

Multiple sclerosis (MS) is a chronic inflammatory, demyelinating and degenerative CNS disease [1]. Pathological changes are fuelled by the activation of circulating and CNS resident immune cells [2]. T, B and natural killer lymphocytes contribute to MS pathology, but the exact interplay between these classes of lymphocytes as well as between different T (i.e., CD4+, CD8+, CD3+CD20+ and regulatory T cells) and B (naïve and memory B cells and naïve and memory B regulatory) subsets is not completely unveiled yet [2]. Striking evidence has accumulated demonstrating that in relapsing stages of MS the success of disease modifying therapies (DMT) in halting disease activity relies on the modulating activity on peripheral immune cells [3]. Conversely, in progressive stages, the role of peripheral immune cells is still questioned. Hereby, CNS-compartmentalised inflammation (i.e., the activation of CNS resident immune cells) is thought to be the major driver for undergoing progressive neuronal loss [4]. A recent study highlighted that in animal models of MS, meningeal B cells, responsible for cortical pathology implicated in progressive MS phenotypes, derive from both the calvarial bone barrow and from blood pool, suggesting a dynamic interchange between peripheral and CNS immune cells [5]. In addition, a group of T cells also expressing B cell markers (CD3+CD20 +) was reported to be increased in peripheral blood of progressive MS [6]. Therefore, drugs acting on modulating peripheral immune cells may also contribute to prevent disease progression.

The only two approved drugs for secondary and primary progressive MS (i.e., siponimod and ocrelizumab) act as immunosuppressors with siponimod preventing lymphocytes egress from secondary lymphoid organs and ocrelizumab depleting circulating B lymphocytes through a CD20-antibody-dependent cellular cytotoxicity [7]. Longitudinal assessment of lymphocytes subset in progressive MS patients treated with siponimod or ocrelizumab, and their correlation with clinical outcomes would provide a window into MS pathogenesis, especially for those mechanisms underpinning neuronal loss. A preliminary analysis assessing lymphocytes changes in siponimod-treated secondary progressive MS patients has been already performed on a relatively small sample from the EXPAND trial with a 1-year follow-up [8]. In this study, authors demonstrated that patients treated with siponimod had reduced overall lymphocyte absolute number and reduced CD4+and CD8+ T cells, as well as increased T and B regulatory cells [8]. However, these findings deserve further confirmation given the relatively small sample size (23 patients) and the short follow-up (1 year). In addition, Wu and colleagues did not assess correlation between lymphocyte changes and clinical outcomes (i.e., relapse occurrence and disability accrual) thus, leaving open questions on the clinical relevance of biological changes.

Against this background, we assessed longitudinal changes of immune cells in patients treated with siponimod over 2-year follow-up time. We aimed to investigate the temporal dynamics of lymphocyte changes in active secondary progressive MS (aSPMS) patients treated with siponimod. We also aimed to explore whether worse clinical outcomes (i.e., disability progression or treatment drop-out) associated with specific trajectories in immune cell changes over time. In line with previous reports [5, 6, 8], we anticipated overall decreased number of naïve T and B cells, an increased number of regulatory B and T cells as well as a reduced rate of circulating CD3+CD20+ cells. We also hypothesised that patients with worse clinical outcomes might present with reduced or absent aforementioned changes.

## Methods

### Study design and population

This was a mono-centric exploratory longitudinal study. We included consecutive aSPMS subjects enrolled at MS Clinical Care and Research Centre of the Federico II University Hospital of Naples, Italy, satisfying the following inclusion criteria: (1) MS diagnosis according to the 2017 McDonald criteria [9] and active progressive phenotype [10]; (2) patients starting on siponimod treatment as for European prescription indication; (3) no history of significant medical illnesses, fever or substance abuse in the 30 day before sample collection; (4) no other major systemic, psychiatric or neurological diseases; (5) no relapse or corticosteroid treatment in the 30 day before sample collection; (6) at least 6 months of follow-up under siponimod treatment and two visits over the follow-up. We also included healthy subjects performing sample collection only at baseline.

### Standard protocol approvals, registrations, and patient consents

Approval was received from the local ethical committees. All subjects gave written informed consent prior to study participation. The study was performed in accordance with good clinical practices and the Declaration of Helsinki.

### Clinical assessment

Patients were followed-up up to 2 years after siponimod start and samples were collected retrospectively and according to scheduled routine clinical visit at baseline, and after 3, 6, 12, and 24 month. At baseline controls also had blood draws.

At baseline we recorded demographic, clinical and radiological data (i.e., age, sex, disease duration [time from symptom onset to baseline visit], previous DMT [platform vs. highly effective treatment as well as treatment classified according to the mechanism of action], CYP2C9 genotype [determining siponimod dosage], time from conversion to aSPMS phenotype, MRI status [new T2 hyperintense lesions, enlarging T2 hyperintense lesions, gadolinium enhancing lesions] and number of previous relapses before siponimod start) as for clinical practice. At baseline and after 3, 6, 12, and 24 month, aSPMS patients underwent a clinical examination, including the assessment of physical disability through the Expanded Disability Status Scale (EDSS) [11]. EDSS progression was defined as sustained increase in EDSS by 1 point if baseline EDSS was 5.5 or lower, or increase in EDSS by 0.5 point if baseline EDSS was above 5.5, assessed 3 months apart [12]. Relapse occurrence was recorded at each study visit and possible corticosteroid treatment in the last 30 days determined study exclusion. Safety information was recorded as for clinical practice.

### Blood sample assessment

At each timepoint, MS patients underwent blood draws. An aliquot (50 μL) of anti-coagulated ethylenediaminetetraacetic acid (EDTA) whole fresh blood (within 12 h) was incubated at 4 °C for 30 min in the presence of appropriate amounts of monoclonal antibodies. The mixtures were then diluted 1:20 in ammonium chloride lysing solution, incubated at room temperature for 10 min and finally washed. Samples were analysed on Becton Dickinson Facs Canto II cytometer BD Facs Diva software. The lower level of detection was 10–4 (as such, zero corresponds to a level below 1/10,000 cells). The values have been expressed both as a percentage and absolute numbers at each time point. For the time-points following baseline we also calculated the percentage change of the absolute number as the ratio between difference between follow-up and baseline measure over baseline measure. If follow-up assessment is zero, we calculated the percentage of the difference between follow-up and baseline assessment. For lymphocyte absolute count, we coupled cytometry to complete blood count on haematological counter (double platform).

The following antigens were analyzed: CD3 Pacific Blu (from Beckman Coulter, Marseille Cedex 9, France), CD4 PEcy5 (from Beckman Coulter, Marseille Cedex 9, France), CD8 APCcy7 (from Beckman Coulter, Marseille Cedex 9, France), CD19 APC (from Beckman Coulter, Marseille Cedex 9, France), CD20 FITC (from Beckman Coulter, Marseille Cedex 9, France), CD56 PEcy7 (from Beckman Coulter, Marseille Cedex 9, France), CD45 FITC (from BD San Diego, CA, USA), CD27 FITC (from BD San Diego, CA, USA), CD24 APC (from Sony Biotechnology, San Jose, CA, USA), CD38 APC (from BD San Diego, CA, USA), CD 127 FITC (from Miltenyi Biotec, Bergisch Gladbach, Germany), CD25 PE (from Miltenyi Biotec, Bergisch Gladbach, Germany), CD45RA APC (from BD San Diego, CA, USA), CD45RO PEcy7 (from Sony Biotechnology, San Jose, CA, USA), CD183 PE (from BD San Diego, CA, USA), CD196 PEcy7 (from BD San Diego, CA, USA) HLA-DR HV500 (from BD San Diego, CA, USA). The lower level of detection was 10–4 (as such, zero corresponds to a level below 1/10,000 cells). The gating strategy was as follows: lymphocyte cells were gated using CD45 vs. SSC-A identifying 50,000 events. This gate was used to identify T lymphocytes (CD3 +), B lymphocytes CD19+ and CD20+ and natural killer NK lymphocytes CD56+CD3− T helper (TH) and T cytotoxic cells were identified as CD3+CD4+ and CD3+CD8+, respectively. CD3+CD20dim represent a heterogeneous T-cell subpopulation. T regulatory cells were identified as CD3+CD4+CD25+CD127−; T-Reg Naive as CD3+CD4+CD25+CD127−CD45RA+ and T-Reg Memory as CD3+CD4+CD25+CD127−CD45RO+. From the lymphocytes (CD45vsSSC-A), activated T lymphocytes were identified as CD3+DR+. B-Reg Naïve cells were identified as CD38+, CD19+ and CD24+ and B-Reg Memory cells were identified as CD38+, CD19+ and CD27+. Laboratory procedures were performed in accordance with UK-NEQAS quality standards (https://ukneqas.org.uk/).

### Statistical analysis

Statistical analyses were performed using the Stata software (version 13; StataCorp LP, College Station, TX). Demographic, clinical and laboratory features of study subjects are presented as means, medians or proportions as appropriate. All demographic, clinical and laboratory variables were checked for normality using the Shapiro–Wilk normality test. Differences between controls and patients for demographic features were assessed through *t* Test, Mann–Whitney *U* or Chi-squared as appropriate. Differences between controls and aSPMS patients for laboratory measures were assessed using logistic regression models adjusted for age and sex.

Changes in laboratory variables over time were explored through generalised linear mixed-effect regression models including laboratory variables, in turn, as dependent variable, timepoint as independent variable (using baseline values as reference), age, sex, genotype and previous DMT category as covariates, and subject id as random factor. Association between laboratory changes over time and clinical outcomes (i.e., EDSS progression or relapse occurrence) were explored using generalised linear mixed-effect regression models including laboratory variables, in turn, as dependent variable, interaction between timepoints and clinical outcomes (EDSS progression, relapse occurrence) as independent variable (using baseline values as reference), age, sex, genotype, follow up time and DMT category as covariates, and subject id as random factor.

A *p* value < 0.05 was considered statistically significant. Given the exploratory nature of the study, no correction for multiple comparisons was applied.

## Results

### Clinical and laboratory measures at baseline

**Fig. 1:**
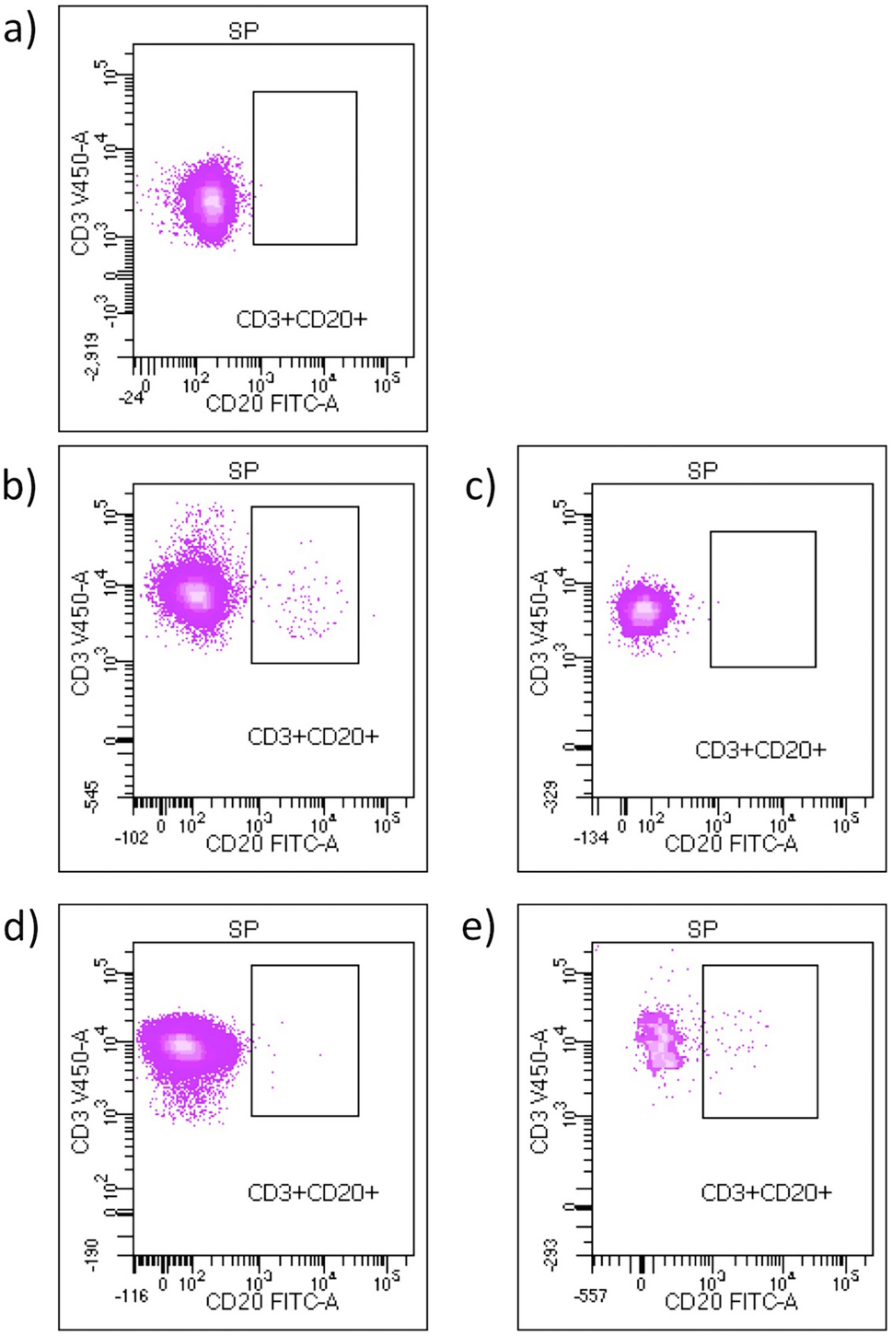
Flow cytometer dot plots. Plot of CD3+CD20+ cells in one male healthy control with no detectable cells (**a**), an aSPMS patient not progressing while on siponimod treatment at baseline with 0.9% CD3+CD20+ cells (**b**) and after 6 months from treatment start with 0.1% CD3+CD20+ cells (**c**) and an aSPMS patient progressing while on siponimod treatment at baseline with 0.1% CD3+CD20+ cells (**d**) and after 6 months from treatment start with 2% CD3+CD20+ cells (**e**)

Demographic, clinical and radiological data from subjects enrolled in the study are summarized in Table 1. We included 46 MS patients and 14 age and sex matched healthy controls. Compared with controls, aSPMS patients presented reduced T lymphocytes (57.35 ± 18.06 vs. 68.29 ± 10.45, *p* = 0.05), increased naïve regulatory T lymphocytes (0.18 ± 0.31 vs. 0.03 ± 0.05; *p* = 0.02), and a trend towards an increase in CD3+CD20+ lymphocytes (0.32 ± 0.63 vs. 0 ± 0; *p* = 0.07). Of note, none of the controls had CD3+CD20+ cells, while 29 out of 46 patients (64%) presented detectable CD3+CD20+ cells at baseline (Fig. 1a, b, d). Results of lymphocyte analysis for patients treated with siponimod and HCs at baseline are outlined in Table 2. Notably, 13 out of 46 patients were previously treated with Anti-CD20 (12 patients treated with rituximab and 1 patient treated with ocrelizumab) with a mean time from last infusion of 15.1 ± 7.1 months. Except for the only patient switching from ocrelizumab to siponimod for tolerability issues and MRI evidence of disease activity (i.e., one enlarging T2 lesion) after 8 months, remaining patients were all refusing infusive treatments and experienced disease progression in the time between anti-CD20 stop and siponimod start. Among patients previously treated with anti-CD20, three patients previously treated with rituximab experienced further disability progression after 7, 8 and 12 months.

**Table 1:** Demographic and clinical features of controls and patients with multiple sclerosis (MS)

	MS patients	HCs	*p* value*
Number of subjects	46	14	
*Sex*			
Male, *N* (%)	18 (39)	4 (29)	0.47
Female, *N* (%)	28 (61)	10 (71)	
Age, mean (SD) (years)	53.6 (6.4)	49.8 (6.2)	0.06
Annualized relapse rate at siponimod start, mean (SD)	0.46 (0.36)	–	
EDSS at siponimod start, median (range)	6 (3–6.5)	–	
Disease duration, median (range) (years)	13 (1–40)	–	
Baseline MRI activity**			
New T2 hyperintense lesions, *N* (%)	16 (41)		
Enlarging T2 hyperintense lesions, *N* (%)	23 (59)		
Gadolinium-enhancing lesions, *N* (%)	0 (0)		
Follow-up time, median (range) (months)	18 (6–24)	–	
*Previous DMT category*			
Platform treatment, *N* (%)	25 (54)	–	
Glatiramer acetate, *N* (%)	4 (9)		
Interferon, *N* (%)	9 (20)		
Dimethyl fumarate, *N* (%)	8 (17)		
Teriflunomide, *N* (%)	4 (9)		
High efficacy treatment, *N* (%)	20 (44)	–	
S1P receptor modulators, *N* (%)	7 (15)		
Anti-CD20, *N* (%)	13 (28)		
Time since last anti-CD20 infusion, mean (SD) (months)	15.51 (7.14)		
Naive, *N* (%)	1 (2)	–	
*Genotype*			
1/1, *N* (%)	32 (70)	–	
1/2, *N* (%)	8 (17)	–	
2/2, *N* (%)	3 (7)	–	
1/3, *N* (%)	2 (4)	–	
2/3, *N* (%)	1 (2)	–	

*MS* multiple sclerosis, *HCs* healthy controls, *EDSS* expanded disability status scale, *SD* standard deviation

^*^ Chi-squared or *t* test as appropriate (*p* < 0.05)

^**^ Data available for 39 patients

**Table 2:** Lymphocytes percentage in multiple sclerosis (MS) patients and healthy controls (HCs)

	MS patients	HCs	*p* value
Number of subjects	46	14	
T lymphocyte, mean ± SD	57.35 ± 18.06	68.29 ± 10.45	0.05*
B lymphocytes, mean ± SD	9.85 ± 6.76	13.5 ± 5.37	0.13
CD4+ lymphocytes, mean ± SD	36.72 ± 16.38	42.36 ± 10.3	0.26
CD8+ lymphocytes, mean ± SD	16.91 ± 6.73	20.29 ± 6.5	0.16
CD4/CD8 ratio, mean ± SD	2.46 ± 1.49	2.3 ± 0.92	0.83
Natural killer lymphocytes, mean ± SD	14.61 ± 11.31	11.07 ± 5.5	0.30
CD3+CD20+ lymphocytes, mean ± SD	0.32 ± 0.63	0 ± 0	0.07
Naïve regulatory T cells, mean ± SD	0.18 ± 0.31	0.03 ± 0.05	0.02*
Memory regulatory T cells, mean ± SD	0.7 ± 0.45	0.61 ± 0.38	0.38
Naïve regulatory B cells, mean ± SD	0.52 ± 0.71	0.36 ± 0.32	0.34
Memory regulatory B cells, mean ± SD	1.17 ± 1.54	1.43 ± 1.89	0.51

*MS* multiple sclerosis, HCs healthy controls, *SD* standard deviation

^*^ Logistic regression adjusted for age and sex (*p* < 0.05)

### Laboratory measures analysis over the follow-up

Results of lymphocyte analysis over the follow-up for patients treated with siponimod are outlined in Table 3. Compared with baseline, aSPMS patients treated with siponimod showed reduced lymphocytes, T lymphocytes, CD4+ lymphocytes, CD4/CD8 ratio, B lymphocytes, memory regulatory B cells from month 3 thereafter (lymphocytes: coeff. range = −699/−486, 95% CI range = −941.08 to −4.55; T lymphocytes: coeff. range = −21/−9, 95% CI range = −29.43 to −1.56; CD4+ lymphocytes: coeff. range = −24/−17, 95% CI range = −31.60 to −10.40; CD4/CD8 ratio: coeff. range = −4.44/−0.67, 95% CI range = −1.61 to −0.17; B lymphocytes: coeff. range = −3.77/−2.54, 95% CI range = −6.02 to −0.35; memory regulatory B: coeff. range = −0.78/−0.57, 95% CI range = −1.24 to −0.17), reduced naïve regulatory T cells from month 6 thereafter (coeff. range = −0.16/−0.11, 95% CI range = −0.27 to −0.02) and reduced CD3+CD20+ lymphocytes from month 12 thereafter (coeff. range = −0.32/−0.24, 95% CI range = −0.59 to −0.03). Conversely, compared with baseline, aSPMS patients treated with siponimod showed increased natural killer lymphocytes from month 3 thereafter (coeff. range = 13.76/19.36, 95% CI range = 6.85–26.45) and increased naïve regulatory B cells from month 6 thereafter (coeff. range = 0.85/1.48, 95% CI range = 0.30–2.18). Finally, aSPMS patients treated with siponimod showed increased CD8+ lymphocytes at month 3 (coeff. = 5.44, 95% CI = 1.89–9.01) and increased memory regulatory T cells at month 6 (coeff. = 0.31, 95% CI = 0.02–0.59). Lymphocyte changes over the follow-up adjusted for the previous DMT classified according to mechanism of action were overlapping (Supplementary Table 1). Moreover, similar results were obtained when expressing lymphocyte subset changes as percentage compared to baseline, (Supplementary Table 2), except for NK cells that did not change over time, for CD3+CD20+ lymphocytes that showed a more pronounced reduction from month 6 thereafter, and for naïve regulatory B cells that only increased at month 24.

**Table 3:** White blood cell count and lymphocytes changes over the follow-up in patients treated with siponimod

	Month 0	Month 3	Coeff.	95% CI		Month 6	Coeff.	95% CI		Month 12	Coeff.	95% CI		Month 24	Coeff.	95% CI	
Number of patients	46	19				27				32				14			
Lymphocytes, mean ± SD	1029 ± 689	558 ± 372	−482.19	−696.34	−268.05	417 ± 225	−566.57	−754.63	−378.51	359.98 ± 153.64	−653.90	−831.59	−476.22	300 ± 83	−705.66	−946.35	−464.97
T lymphocyte, mean ± SD	57.35 ± 18.06	48.74 ± 13.68	−8.86	−16.17	−1.56	38.48 ± 16.25	−17.25	−23.63	−10.87	38.34 ± 18	−18.67	−24.68	−12.66	35.93 ± 14.5	−21.18	−29.43	−12.94
B lymphocytes, mean ± SD	9.85 ± 6.76	4.76 ± 3.15	−3.77	−6.02	−1.53	7.3 ± 5.5	−2.56	−4.52	−0.60	7 ± 5.61	−2.513	−4.35	−0.67	7.43 ± 6.44	−2.89	−5.44	−0.35
CD4+ lymphocytes, mean ± SD	36.72 ± 16.38	19.37 ± 11.77	−16.87	−23.31	−10.43	18.52 ± 10.34	−18.10	−23.95	−12.41	15 ± 9.36	−21.65	−27.05	−16.26	12.64 ± 7.08	−24.36	−31.57	−17.15
CD8+ lymphocytes, mean ± SD	16.91 ± 6.73	23.53 ± 9.57	5.47	1.91	9.03	15.74 ± 8.51	−0.25	−3.35	2.85	16.94 ± 11.07	−0.32	−3.25	2.59	18 ± 11.22	1.02	−3.00	5.05
CD4/CD8 ratio, mean ± SD	2.46 ± 1.49	1.08 ± 1.3	−1.02	−1.60	−0.44	1.75 ± 1.71	−0.68	−1.17	−0.18	1.24 ± 1.23	−1.11	−1.61	−0.61	1.21 ± 1.82	−4.36	−6.55	−2.17
NK lymphocytes, mean ± SD	14.61 ± 11.31	29.47 ± 17.25	13.92	6.84	21.00	28.69 ± 14.19	13.75	7.48	20.02	34.22 ± 19.62	19.35	13.51	25.18	33.36 ± 20.16	18.47	10.49	26.45
CD3+CD20+ lymphocytes, mean ± SD	0.32 ± 0.63	0.12 ± 0.24	−0.19	−0.43	0.06	0.11 ± 0.41	−0.20	−0.42	0.02	0.08 ± 0.44	−0.24	−0.45	−0.03	0 ± 0	−0.32	−0.59	−0.04
Naïve regulatory T cells, mean ± SD	0.18 ± 0.31	0.08 ± 0.15	−0.1	−0.20	0.002	0.07 ± 0.1	−0.12	−0.21	−0.02	0.03 ± 0.07	−0.16	−0.24	−0.07	0.03 ± 0.06	−0.15	−0.27	−0.04
Memory regulatory T cells, mean ± SD	0.7 ± 0.45	0.59 ± 0.58	−0.11	−0.43	0.20	1.01 ± 0.81	0.30	0.02	0.58	0.79 ± 0.71	0.09	−0.18	0.36	0.54 ± 0.35	−0.16	−0.51	0.19
Naïve regulatory B cells, mean ± SD	0.52 ± 0.71	0.63 ± 0.49	0.28	−0.33	0.90	1.33 ± 1.16	0.84	0.30	1.38	1.53 ± 1.7	1.03	0.51	1.54	1.98 ± 2.32	1.49	0.80	2.18
Memory regulatory B cells, mean ± SD	1.17 ± 1.54	0.41 ± 0.62	−0.57	−0.97	−0.17	0.49 ± 0.65	−0.63	−1.00	−0.28	0.47 ± 0.8	−0.61	−0.94	−0.28	0.41 ± 0.53	−0.78	−1.24	−0.33

Coefficients and 95% CI were obtained through age-, sex-, genotype and DMT category-corrected generalized linear mixed-effect regression models using baseline values as reference

*CI* confidence interval, *NK* natural killer, *SD* standard deviation

### Association between laboratory changes and clinical outcomes

Over the follow-up, only one patient experienced a clinical relapse, therefore, the association between lymphocyte changes and relapse occurrence was not assessed. Ten patients (22%) dropped from siponimod treatment after a mean time of 18 ± 8 months. Five patients dropped because of EDSS progression, four patients dropped because of adverse events (two patients presented with persistent migraine, one patient presented with grade III lymphopenia and one patient presented with hypertension) and one patient dropped for personal decision. Dropped-out patients did no longer perform blood sample. Disability progression was observed in 10 patients (22%) after a mean follow-up of 9.3 ± 2.7 months. Results for the association between disability progression and laboratory trajectories are depicted in Table 4.

**Table 4:** Association between longitudinal lymphocytes and disability progression in multiple sclerosis patients treated with siponimod

	Month 3			Month 6			Month 12			Month 24		
	Coeff.	95% CI		Coeff.	95% CI		Coeff.	95% CI		Coeff.	95% CI	
Lymphocytes												
Non progressing	−502.83	−742.51	−263.15	−600.45	−809.16	−391.74	−636.80	−842.64	−430.95	−640.34	−957.37	−323.31
Progressing	−419.31	−877.45	38.83	−380.42	−801.75	40.90	−646.08	−993.03	−299.12	−675.31	−1071.52	−279.09
B lymphocytes												
Non progressing	−3.715	−6.23	−1.20	−2.28	−4.44	−0.11	−2.86	−4.99	−0.73	−4.22	−7.52	−0.93
Progressing	−3.87	−8.65	0.91	−4.34	−8.73	0.04	−1.83	−5.38	1.72	−1.23	−5.33	2.86
CD8+ lymphocytes												
Non progressing	5.40	1.45	9.36	0.69	−2.72	4.11	−0.06	−3.43	3.30	−0.59	−5.78	4.61
Progressing	5.12	−2.42	12.65	−3.95	−10.86	2.96	−1.03	−6.64	4.58	2.85	−3.61	9.31
CD4+ lymphocytes												
Non progressing	−15.70	−22.93	−8.47	−16.73	−23.07	−10.40	−20.03	−26.28	−13.78	−22.45	−32.04	−12.86
Progressing	−20.61	−34.42	−6.79	−23.28	−36.02	−10.55	−26.20	−36.79	−15.60	−27.84	−39.86	−15.81
CD4/CD8 ratio												
Non progressing	−1.20	−1.81	−0.58	−0.80	−1.33	−0.26	−1.05	−1.58	−0.53	−1.45	−2.27	−0.64
Progressing	−1.10	−2.28	0.08	−0.72	−1.80	0.36	−1.30	−2.19	−0.43	−1.56	−2.58	−0.55
Natural killer lymphocytes												
Non progressing	11.85	3.79	19.92	13.78	6.79	20.78	15.68	8.79	22.57	22.62	11.98	33.26
Progressing	12.17	−3.24	27.57	14.00	−0.14	28.15	22.55	10.99	34.10	12.31	−0.95	25.57
CD3+CD20+ lymphocytes												
Non progressing	−0.17	−0.45	0.10	−0.29	−0.53	−0.05	−0.23	−0.47	0.004	−0.39	−0.75	−0.02
Progressing	−0.20	−0.72	0.32	0.12	−0.36	0.60	−0.29	−0.69	0.12	−0.31	−0.77	0.15
Naïve regulatory T cells												
Non progressing	8.71	2.61	14.81	8.38	3.08	13.69	11.80	6.57	17.03	8.95	0.89	17.01
Progressing	9.60	−2.06	21.26	6.02	−4.69	16.74	9.15	0.36	17.95	3.58	−6.48	13.64
Memory regulatory T cells												
Non progressing	−7.64	−13.52	−1.76	−9.02	−14.12	−3.92	−10.03	−15.06	−5.00	−5.32	−13.08	2.43
Progressing	−10.88	−22.11	0.35	−10.76	−21.07	−0.44	−7.11	−15.55	1.33	−0.24	−9.91	9.43
Naïve regulatory B cells												
Non progressing	0.21	−0.48	0.89	0.86	0.26	1.45	0.91	0.31	1.51	0.90	−0.006	1.81
Progressing	0.55	−0.76	1.86	0.56	−0.65	1.76	1.29	0.30	2.28	2.26	1.13	3.39
Memory regulatory B cells												
Non progressing	−0.54	−0.99	−0.09	−0.61	−1.00	−0.22	−0.64	−1.03	−0.25	−0.60	−1.19	−0.01
Progressing	−0.67	−1.53	0.18	−0.70	−1.48	0.09	−0.51	−1.15	0.13	−0.97	−1.70	−0.23

Coefficients and 95% CI were obtained through age-, sex-, genotype and DMT category-corrected generalised linear mixed-effect regression models using baseline values as reference

Differently from patients experiencing disability progression, patients not experiencing disability progression while on siponimod treatment revealed reduced B lymphocytes from month 3 thereafter (coeff. range = −4.22/−2.28, 95% CI range = −7.52 to −0.11), increased CD8+ T lymphocytes at month 3 (coeff. = 5.40, 95% CI = 1.45–9.36), reduced CD4/CD8 ratio already at month 3 and 6 (coeff. = −1.20, 95% CI = −1.81 to −0.58; coeff. = −0.80, 95% CI = −1.33 to −0.26; respectively), increased natural killer already at month 3 and 6 (coeff. = 11.85, 95% CI = 3.79–19.92; coeff. = 13.78, 95% CI = 6.79–20.78 respectively), reduced CD3+CD20+ lymphocytes at month 6 (coeff. = −0.29, 95% CI = −0.53 to −0.05), increased naïve regulatory B cells already at month 6 (coeff. = 0.86, 95% CI = 0.26–1.45), and reduced memory regulatory B cells from month 3 thereafter (coeff. range = −0.64/−0.54, 95% CI range = −1.03 to −0.09). When assessing percentage change of the absolute number we confirmed that not progressing MS patients showed a marker reduction of CD3+CD20+ lymphocytes from month 6 thereafter, a slight naïve regulatory T cells reduction from month 6 thereafter and a marked reduction in memory regulatory B cells from month 3 thereafter with no differences among progressing and non-progressing patients for CD8+ T lymphocytes, CD4/CD8 ratio and natural killer (Supplementary Table 3).

## Discussion

In this study, we investigated peripheral lymphocyte changes over time in aSPMS patients treated with siponimod and their associations with disability progression. aSPMS patients presented with an overall T cell reduction with increased naïve regulatory T lymphocytes and a trend towards an increase in CD3+CD20+ lymphocytes. Patients treated with siponimod showed a sustained reduction of T lymphocytes, especially CD4+, CD3+CD20+ and naïve regulatory T cells, B lymphocytes and memory regulatory B cells, with relative increase of naïve regulatory B lymphocytes. In addition, we showed that disability progression while on siponimod treatment was associated with the lack of drug effect on B lymphocytes and CD3+CD20+ lymphocytes.

Previous studies demonstrated that T lymphocyte subpopulations are altered in MS compared with healthy controls, but characterizations of T-cell subset are discordant. In some studies, MS patients showed increased levels of CD8+ effector T-cells in peripheral blood [13], while other authors described a decreased number of this T-cell subset [14, 15] and the reason for this discrepancy might lay in the different MS phenotypes included in these studies. SPMS and primary progressive MS patients showed normal or increased frequency of effector and memory CD8+ T-cells [15, 16] vs. controls and relapsing–remitting MS. Differently from T lymphocytes subsets that have been widely studied, a full characterization of B-cell subpopulations in peripheral blood of MS patients is still lacking [17] and, only recently, with the introduction of CD20-targeting drugs, this cell population is under investigation. DMTs in MS act by modulating patients’ lymphocytes with different mechanisms of action. Previous studies evaluating the impact of different treatments on immune cells have demonstrated a peculiar immunomodulatory profile for each drug. For example, dimethyl fumarate showed increased percentage of naïve and effector T cells [18, 19], natural killer [19] and naïve B cells [19], though the total B cell count decreased especially for memory cells [20]. Conversely, by impeding lymphocytes from crossing the blood–brain barrier, natalizumab produces an increased count for total lymphocytes, natural killers, CD8+, memory and regulatory B cells [21, 22] and increased B cell percentage [23]. With specific regards to sphingosine-1 receptor modulators, most of available findings refer to the effect of fingolimod. These findings report on decreased naïve and memory B cells with increased regulatory B cells [23–25], decreased CD4+ T cells [23–25], and decreased CD8+ T cells [25], whilst no changes were detected for natural killer lymphocytes [25, 26]. The only available report on siponimod highlights that patients have a reduction of B cells, CD4+ and CD8+ T cells, with an increase of regulatory B cells, which is quite in line with our finding supporting the reduction of T cells lineage and an increase in the regulatory cells [8]. Taken together, these reports suggest that while DMTs for MS have the common goal to prevent disease activity (relapse and disability progression), the regulation of the immunological cells is specific for each drug. Similarly to fingolimod, siponimod produces a shift toward a more regulatory environment mediated by B cells and a prevalent reduction of the T cell lineage.

Another interesting finding from our study is the association between disability progression while under siponimod treatment and the trajectory of lymphocytes changes over time. Specifically, we reported that patients presenting with disability progression showed a reduced effect of siponimod on B cells, CD3+CD20+ lymphocytes and naive regulatory B cells.

While the association with the lack of effect on B cells in patients with disability accrual was quite expected, given that the only other drug showing efficacy in halting disability progression in MS is the anti-CD20 ocrelizumab [27], the effect on CD3+CD20+ cells is quite intriguing and novel. CD3+CD20+ cells derive from the trogocytosis occurring during the B mediated-T cell activation process, which is when B and T cells get in contact for the activation of T cells, these latter steal part of the B cell membrane, thus co-expressing CD20 antigen on their surface [28].

Some evidence showed that CD3+CD20+ cells have pro-inflammatory activity and could play a role in pathogenesis of autoimmune disorders. For example, higher levels were found in psoriasis, rheumatoid arthritis and Sjogren’s disease [29]. Previous reports have already demonstrated that in patients with MS, higher levels of CD3+CD20+ cells are associated with an up-regulation of production of proinflammatory cytokines, such as IFN-γ, GM-CSF, IL-17, and TNFα [30]. Moreover, CD3+CD20+ cells are present in blood and chronic brain lesions of MS patients [31], and their level was higher in PPMS compared with healthy controls, correlating with EDSS [6]. This evidence indicated that CD3+CD20+ cells may play a role in progression of MS and hence they deserve to be further investigated.

T and B regulatory cells appear to sustain immune tolerance, through production of anti-inflammatory cytokines (e.g., IL-10), but their role in MS pathogenesis is still poorly understood [32].

In patients with MS, T regulatory cells are found to be increased in the CSF [33], but not in peripheral blood. However, RRMS patients showed reduced naïve T regulatory cells in favor of memory T regulatory [34], whilst in progressive stages of disease, T regulatory cells showed recovery of their normal function [35].

On the other hand, contradictive reports have been published about B regulatory cells levels in MS patients [36]. However, B regulatory cells in MS patients showed a reduction in production of IL-10 compared to controls [37].

DMTs seem to affect these subpopulations in a peculiar way. Fingolimod, for example, affects regulatory lymphocyte populations, as it leads to increase of both B and T regulatory cells [38, 39]. Similarly, in our study siponimod induced a significant change in B and T regulatory populations in aSPMS patients, in particular an increasing of naïve regulatory B cells and memory regulatory T cells. Moreover, patients experiencing disability progression while on siponimod treatment did not show increase in naïve regulatory B cells and reduction in memory regulatory B cells. Although these results point towards a role of these cells in progression of disease, more studied are needed to confirm our finding.

Although in our study we mostly focused on the association between lymphocyte changes and overt disability accrual, it would be interesting also to explore the association between lymphocyte changes and other biological and clinical factors associated with progression. For example, it would be intriguing to explore the association between lymphocyte changes, especially those relevant for the EDSS progression and invisible symptoms associated with progression such as fatigue, cognition and depressive symptoms. Furthermore, it would be even more interesting to evaluate the association between lymphocyte changes and advanced MRI metrics such as paramagnetic rim lesion and slowly expanding lesions. Previous studies have clearly demonstrated the association between paramagnetic rim lesion and slowly expanding lesions with EDSS progression, and the predictive value of these metrics [40–44]. In our study we could not assess such an association, nor we could exclude the occurrence of overt MRI inflammation during follow-up, since we did not properly collect MRI scans in a standardised fashion. Therefore, future studies could be able to assess the presumptive association further emphasizing the possible predictive value of lymphocytes subset related to disability accrual.

We do acknowledge that this study is not without limitations. First, whilst this is a longitudinal study, patients did not strictly adhere to the study timelines resulting in different numbers of patients at different timepoints. However, to counteract this limitation we modelled trajectories using a mixed-linear statistical model. Secondly, we did not collect any MRI finding which could be helpful to further elucidate the role of lymphocyte populations on different pathological processes underpinning MS. Thirdly, sample size for controls is quite limited and we only obtained laboratory sample at baseline in this population, thus possibly limiting the assessment of lymphocyte changes in siponimod-treated patients. However, lymphocyte changes were assessed against baseline thus accounting for possible inter-subjects variability.

Finally, patients undergoing siponimod treatment may have received previous DMTs that could also impact on lymphocyte subsets, thus biasing our results. However, we used DMT category as covariate to account for this possible bias.

Future multicentre studies with higher sample size are needed to investigate lymphocyte changes in aSPMS while on siponimod treatment, eventually using healthy subjects, aSPMS, non-active SPMS or SPMS patients not taking DMT as the comparator.

In conclusion, in our study, we observed that patients with aSPMS treated with siponimod had decreased number of both naïve T and B cells, an increased number of regulatory B and T cells, as well as a reduced rate of circulating CD3+CD20+ cells over 2-year follow-up. Moreover patients that experienced disability progression while on siponimod treatment did not demonstrate reduction in B lymphocytes and CD3+CD20+ lymphocytes. Therefore, the analysis of lymphocyte subpopulations in peripheral blood in MS patients could be a biomarker for treatment efficacy, especially in those patients with progressive disease course.

### Supplementary Information

Below is the link to the electronic supplementary material.

Supplementary file1 (DOCX 33 KB)

## Data Availability

The anonymised dataset used and analyzed during the current study is available from the corresponding author upon reasonable request.
