# Supplementary material for: Effect of siponimod on lymphocyte subsets in active secondary progressive multiple sclerosis and clinical implications
Source: J Neurol. 2024 Apr 17;271(7):4281–91. doi: 10.1007/s00415-024-12362-9 (PMC11233419; doi:10.1007/s00415-024-12362-9)
Supplement: Supplementary file 1 — Supplementary file1 (DOCX 33 KB) [file 415_2024_12362_MOESM1_ESM.docx]

**Effect of siponimod on lymphocyte subsets in active secondary progressive multiple sclerosis and clinical implications**

**Journal name: Journal of Neurology**

Antonio Luca Spiezia^1^, Giulia Scalia^2^, Maria Petracca^3^, Daniele Caliendo^1^, Marcello Moccia^4^, Antonia Fiore^2^, Vincenza Cerbone^2^, Roberta Lanzillo^1^, Vincenzo Brescia Morra^1^, Antonio Carotenuto^1^.

^1^Multiple Sclerosis Clinical Care and Research Centre, Department of Neuroscience, Reproductive Science and Odontostomatology, Federico II University of Naples, Naples, Italy; ^2^Laboratorio di Citometria Clinica e Sperimentale CEINGE-Biotecnologie Avanzate Franco Salvatore; ^3^Department of Human Neurosciences, Sapienza University, Rome, Italy; ^4^Department of Molecular Medicine and Medical Biotechnology, Federico II University of Naples, Italy.

**Correspondence should be addressed to**: Antonio Carotenuto, Multiple Sclerosis Clinical Care and Research Centre, Department of Neuroscience, Reproductive Science and Odontostomatology Federico II University, Via Sergio Pansini 5, 80131 Naples, Italy. Telephone number: +39 0817462670; Fax number: +39 0817462670; E-mail address: carotenuto.antonio87@gmail.com

**Supplementary Table 1. White blood cell count and lymphocytes changes over the follow-up in patients treated with siponimod.** Coefficients and p values were obtained through age-, sex-, genotype and DMT category according to mechanism of action-corrected generalised linear mixed-effect regression models using baseline values as reference.

|  | **Month 0** | **Month 3** | **coeff.** | **pvalue** | **Month 6** | **coeff.** | **p value** | **Month 12** | **coeff.** | **p value** | **Month 24** | **coeff.** | **p value** |
| --- | --- | --- | --- | --- | --- | --- | --- | --- | --- | --- | --- | --- | --- |
| **Number of patients** | 46 | 19 |  |  | 27 |  |  | 32 |  |  |  |  |  |
| **Lymphocytes, mean ± SD** | 1029 ± 689 | 558 ± 372 | -485.48 | <0.001 | 417 ± 225 | -571.47 | <0.001 | 359.98 ± 153.64 | -651 | <0.001 | 300 ± 83 | -717.05 | <0.001 |
| **T Lymphocyte, mean ± SD** | 57.35 ± 18.06 | 48.74 ± 13.68 | -8.86 | 0.02 | 38.48 ± 16.25 | -17.18 | <0.001 | 38.34 ± 18 | -18.42 | <0.001 | 35.93 ± 14.5 | -21.58 | <0.001 |
| **B Lymphocytes, mean ± SD** | 9.85 ± 6.76 | 4.76 ± 3.15 | -3.7 | 0.001 | 7.3 ± 5.5 | -2.5 | 0.01 | 7 ± 5.61 | -2.43 | 0.009 | 7.43 ± 6.44 | -2.89 | 0.02 |
| **CD4+Lymphocytes, mean ± SD** | 36.72 ± 16.38 | 19.37 ± 11.77 | -17.16 | <0.001 | 18.52 ± 10.34 | -17.97 | <0.001 | 15 ± 9.36 | -21.28 | <0.001 | 12.64 ± 7.08 | -25.39 | <0.001 |
| **CD8+Lymphocytes, mean ± SD** | 16.91 ± 6.73 | 23.53 ± 9.57 | 5.45 | 0.003 | 15.74 ± 8.51 | -0.26 | 0.087 | 16.94 ± 11.07 | -0.32 | 0.83 | 18 ± 11.22 | 0.98 | 0.63 |
| **CD4/CD8 ratio, mean ± SD** | 2.46 ± 1.49 | 1.08 ± 1.3 | -1.18 | <0.001 | 1.75 ± 1.71 | -0.74 | 0.003 | 1.24 ± 1.23 | -1.08 | <0.001 | 1.21 ± 1.82 | -1.51 | <0.001 |
| **NK lymphocytes, mean ± SD** | 14.61 ± 11.31 | 29.47 ± 17.25 | 11.97 | 0.001 | 28.69 ± 14.19 | 13.76 | <0.001 | 34.22 ± 19.62 | 17.64 | <0.001 | 33.36 ± 20.16 | 18.33 | <0.001 |
| **CD3+CD20+ Lymphocytes, mean ± SD** | 0.32 ± 0.63 | 0.12 ± 0.24 | -0.19 | 0.14 | 0.11 ± 0.41 | -0.20 | 0.07 | 0.08 ± 0.44 | -0.25 | 0.02 | 0 ± 0 | -0.30 | 0.03 |
| **Naïve regulatory T cells, mean ± SD** | 0.18 ± 0.31 | 0.08 ± 0.15 | -0.10 | 0.06 | 0.07 ± 0.1 | -0.11 | 0.02 | 0.03 ± 0.07 | -0.15 | 0.001 | 0.03 ± 0.06 | -0.16 | 0.006 |
| **Memory regulatory T cells, mean ± SD** | 0.7 ± 0.45 | 0.59 ± 0.58 | -0.11 | 0.48 | 1.01 ± 0.81 | 0.31 | 0.03 | 0.79 ± 0.71 | 0.10 | 0.49 | 0.54 ± 0.35 | -0.17 | 0.36 |
| **Naïve regulatory B cells, mean ± SD** | 0.52 ± 0.71 | 0.63 ± 0.49 | 0.28 | 0.37 | 1.33 ± 1.16 | 0.83 | 0.002 | 1.53 ± 1.7 | 1.02 | <0.001 | 1.98 ± 2.32 | 1.52 | <0.001 |
| **Memory regulatory B cells, mean ± SD** | 1.17 ± 1.54 | 0.41 ± 0.62 | -0.58 | 0.005 | 0.49 ± 0.65 | -0.64 | <0.001 | 0.47 ± 0.8 | -0.62 | <0.001 | 0.41 ± 0.53 | -0.80 | 0.001 |

Abbreviations: NK, Natural Killer; SD, standard deviation.

**Supplementary Table 2. White blood cell count and lymphocytes changes expressed as percentage change in the absolute number over the follow-up in patients treated with siponimod.** Coefficients and p values were obtained through age-, sex-, genotype and DMT category according to mechanism of action-corrected generalised linear mixed-effect regression models using baseline values as reference.

|  | **Month 3** | | **Month 6** | | **Month 12** | | **Month 24** | |
| --- | --- | --- | --- | --- | --- | --- | --- | --- |
| **Number of patients** | **coeff.** | **p value** | **coeff.** | **p value** | **coeff.** | **p value** | **coeff.** | **p value** |
| **Lymphocytes, mean ± SD** | -36.52 | <0.001 | -39.31 | <0.001 | -51.72 | <0.001 | -60.99 | <0.001 |
| **T Lymphocyte, mean ± SD** | -28.29 | 0.002 | -44.64 | <0.001 | -61.14 | <0.001 | -68.22 | <0.001 |
| **B Lymphocytes, mean ± SD** | -35.93 | 0.03 | -30.72 | 0.04 | -34.66 | 0.01 | -66.43 | 0.001 |
| **CD4+Lymphocytes, mean ± SD** | -30.95 | 0.03 | -40.00 | 0.001 | -63.44 | <0.001 | -76.44 | <0.001 |
| **CD8+Lymphocytes, mean ± SD** | -11.4 | 0.24 | -33.05 | <0.001 | -53.34 | <0.001 | -55.52 | <0.001 |
| **CD4/CD8 ratio, mean ± SD** | -38.03 | 0.002 | -12.24 | 0.26 | -23.15 | 0.03 | -52.71 | <0.001 |
| **NK lymphocytes, mean ± SD** | 24.60 | 0.23 | 23.99 | 0.18 | -1.68 | 0.92 | 4.70 | 0.84 |
| **CD3+CD20+ Lymphocytes, mean ± SD** | -279.25 | 0.09 | -335.06 | 0.009 | -515.71 | 0.001 | -315.59 | 0.06 |
| **Naïve regulatory T cells, mean ± SD** | -118.33 | 0.04 | -90.06 | 0.09 | -124.13 | 0.009 | -260.78 | <0.001 |
| **Memory regulatory T cells, mean ± SD** | 2.19 | 0.95 | 4.46 | 0.88 | -23.85 | 0.39 | -43.16 | 0.25 |
| **Naïve regulatory B cells, mean ± SD** | 75.84 | 0.24 | 59.30 | 0.30 | -13.60 | 0.80 | 146.92 | 0.04 |
| **Memory regulatory B cells, mean ± SD** | -187.45 | 0.02 | -147.18 | 0.04 | -202.84 | 0.01 | -218.42 | 0.02 |

Abbreviations: NK, Natural Killer; SD, standard deviation.

**Supplementary Table 3. Association between longitudinal lymphocytes and disability progression in multiple sclerosis patients treated with siponimod.** Coefficients and p values were obtained through age-, sex-, genotype and DMT category according to mechanism of action-corrected generalised linear mixed-effect regression models using baseline values as reference.

|  |  | **Month 3** | | **Month 6** | | **Month 12** | | **Month 24** | |
| --- | --- | --- | --- | --- | --- | --- | --- | --- | --- |
|  |  | **coeff.** | **p value** | **coeff.** | **p value** | **coeff.** | **p value** | **coeff.** | **p value** |
| **T Lymphocytes** | **Non progressing** | -27.77 | 0.01 | -39.55 | <0.001 | -54,33 | <0.001 | -57.76 | <0.001 |
|  | **Progressing** | -33.49 | 0.09 | -55.64 | 0.002 | -70.97 | <0.001 | -73.51 | <0.001 |
| **B Lymphocytes** | **Non progressing** | -27.99 | 0.007 | -40.40 | <0.001 | -55.58 | <0.001 | -57.19 | <0.001 |
|  | **Progressing** | -31.64 | 0.11 | -54.63 | 0.003 | -70.68 | <0.001 | -73.27 | <0.001 |
| **CD8+ Lymphocytes** | **Non progressing** | -11.59 | 0.29 | -31.08 | 0.001 | -49.24 | <0.001 | -46.33 | 0.001 |
|  | **Progressing** | -13.39 | 0.52 | -34.73 | 0.07 | -59.56 | 0.001 | -59.03 | <0.001 |
| **CD4+ Lymphocytes** | **Non progressing** | -26.65 | 0.09 | -31.27 | 0.03 | -54.38 | 0.001 | -62.35 | 0.003 |
|  | **Progressing** | -49.67 | 0.10 | -64.89 | 0.02 | -80.21 | 0.001 | -79.87 | 0.003 |
| **CD4/CD8 ratio** | **Non progressing** | -38.76 | 0.005 | -5.13 | 0.67 | -9.61 | 0.42 | -40.72 | 0.03 |
|  | **Progressing** | -36.80 | 0.16 | -33.75 | 0.17 | -53.94 | 0.007 | -60.06 | 0.009 |
| **Natural killer lymphocytes** | **Non progressing** | 27.41 | 0.22 | 9.17 | 0.64 | 1.52 | 0.94 | 32.88 | 0.27 |
|  | **Progressing** | 18.13 | 0.67 | 87.92 | 0.03 | -13.01 | 0.69 | -41.83 | 0.26 |
| **CD3+CD20+ Lymphocytes** | **Non progressing** | -340.01 | 0.07 | -377.80 | 0.009 | -489.07 | <0.001 | -422.57 | 0.05 |
|  | **Progressing** | -83.04 | 0.79 | -200.50 | 0.49 | -222.32 | 0.35 | -157.13 | 0.56 |
| **Naïve regulatory T cells** | **Non progressing** | -157.19 | 0.02 | -63.98 | 0.27 | -114.46 | 0.04 | -359.88 | <0.001 |
|  | **Progressing** | 11.65 | 0.92 | -190.96 | 0.08 | -151.12 | 0.09 | -150.9 | 0.14 |
| **Memory regulatory T cells** | **Non progressing** | 21.77 | 0.56 | 26.64 | 0.41 | -9.51 | 0.77 | -23.59 | 0.62 |
|  | **Progressing** | -71.50 | 0.30 | -66.25 | 0.30 | -51.46 | 0.35 | -40.83 | 0.53 |
| **Naïve regulatory B cells** | **Non progressing** | 91.99 | 0.21 | 66.27 | 0.30 | -36.57 | 0.55 | 162.26 | 0.09 |
|  | **Progressing** | 38.60 | 0.77 | -15.15 | 0.91 | 17.41 | 0.87 | 91.18 | 0.46 |
| **Memory regulatory B cells** | **Non progressing** | -234.66 | 0.01 | -176.35 | 0.02 | -268.03 | <0.001 | -234.44 | 0.04 |
|  | **Progressing** | -21.80 | 0.90 | -26.58 | 0.88 | -36.31 | 0.78 | -181.54 | 0.21 |

Abbreviations: NK, Natural Killer; SD, standard deviation
